# Malignant transformation of lower-grade glioma: contrast enhancement, extent of resection, and the natural history under interval-censored analysis

**DOI:** 10.1007/s11060-026-05803-0

**Published:** 2026-09-22

**Authors:** Baran Atli, Maximilian Wartha, Matthias Gmeiner, Martin Aichholzer, Serge Weis, Dominik Vorhauer, Helga Wagner, Tanja Hauer, Carlo Serra, Petra Böhm, Michael Sonnberger, Nico Stroh-Holly, Stefan Aspalter, Kathrin Aufschnaiter, Tobias Rossmann, Maria Gollwitzer, Jawed Ziai, Annette Leibetseder, Josef Pichler, Wolfgang Thomae, Christian Dorfer, Andreas Gruber, Harald Stefanits, Philip Rauch

**Affiliations:** 1 Department of Neurosurgery, Kepler University Hospital, Johannes Kepler University Linz, Neuromed Campus, Wagner-Jauregg-Weg 15, Linz, 4020 Austria; 2 Johannes Kepler University Linz, Linz, Austria; 3 Division of Neuropathology, Department of Pathology and Molecular Pathology, Kepler University Hospital, Linz, Austria; 4 Clinical Research Institute for Neuroscience, Johannes Kepler University, Linz, Austria; 5 Institute of Statistics, Johannes Kepler University, Linz, Austria; 6 Department of Neurosurgery, Clinical Neuroscience Center, University Hospital, University of Zurich, Zurich, Switzerland; 7 Machine Intelligence in Clinical Neuroscience (MICN) Lab, Clinical Neuroscience Center, University Hospital Zurich, University of Zurich, Zurich, Switzerland; 8 Institute of Neuroradiology, Kepler University Hospital, Johannes Kepler University, Linz, Austria; 9 Department of Neurology, Kepler University Hospital, Johannes Kepler University Linz, Linz, Austria; 10 Department of Internal Medicine and Neurooncology, Neuromed Campus, Kepler University Hospital, Linz, Austria; 11 Department of Neurosurgery, University Hospital St. Pölten, Karl Landsteiner University of Health Sciences, St. Pölten, Austria

**Keywords:** Lower-grade glioma, Malignant transformation, IDH stratification, Bayesian interval-censored survival analysis, Radiomics, Extent of resection

## Abstract

**Purpose:**

Malignant transformation (MT) is the principal driver of mortality in lower-grade glioma (LGG), yet its preoperative determinants and timing remain poorly defined. We quantified imaging elements of MT and its timing, correcting for bias in dating transformation.

**Methods:**

We evaluated contrast enhancement (CE), tumour volume and 99 radiomic features in 155 patients with WHO grade 2–3 LGG, radiologically low-grade at diagnosis with at most minimal enhancement (77 transformations; median surveillance among non-transformers 4.8 years). Because MT is ascertainable only between scans, timing was modelled as interval-censored across four complementary models, with extent of resection (EOR) time-varying.

**Results:**

CE at diagnosis was the strongest predictor of MT, exceeding tumour volume and a co-modelled 99-feature radiomic panel (Bayesian HR 22.1, 95% CrI 10.5–53.2); conditioned on transformation-free survival to six months, HR 5.65 (95% CI 2.23–14.31). No radiomic feature survived shrinkage or native interval-censoring. Initial enhancement predicted post-surgical transformation independently of grade, IDH status and EOR, even after gross-total resection (GTR), itself associated with a two-thirds lower hazard (HR 0.32, 95% CI 0.13–0.77). Interval censoring proved material: conventional dating overstated the hazard four-fold.

**Conclusion:**

CE at diagnosis predicted transformation risk and timing. It remained predictive after GTR removed the enhancing tissue, a postoperative role not previously recognised. The signal was present in each integrated tumour type and survived adjustment for grade, IDH status and EOR, may reflect intrinsic tumour biology and could refine postoperative risk estimation. GTR was the only modifiable determinant. These are the first interval-censored estimates of MT timing.

**Supplementary Information:**

The online version contains supplementary material available at https://doi.org/10.1007/s11060-026-05803-0.

## Introduction

Adult-type diffuse gliomas of WHO grade 2 or 3 — lower-grade gliomas (LGGs) — follow a deceptively indolent course before malignant transformation (MT) [1–3], the inflection that drives prognosis, adjuvant-therapy timing and trial eligibility [2, 3]. Preoperative imaging is the only risk marker available before tissue is obtained; molecular classification under the 2021 WHO scheme [4, 5] emerges only at biopsy or resection and thereafter defines the subgroup within which imaging must be read. How strongly imaging predicts transformation within each molecular class remains undefined [6–10]. Timing is equally uncertain: MT is seldom bracketed by two consecutive surveillance MRIs, the only configuration in which it can be localised in time.

Three methodological gaps shape this literature. First, MT has not previously been analysed as an interval-censored event: prior studies date it to the confirmatory MRI and treat it as right-censored, mis-specifying the event time and biasing the estimator [11, 12]. Second, radiomics pipelines yield ≥ 100 candidate features per patient, but published analyses rely on univariate screening or LASSO, both miscalibrated at LGG event counts [13–16]. Third, LGG treatment is dynamic, inducing time-varying state changes that most Cox analyses ignore [17, 18].

In a long-term cohort spanning the biological dynamic range of LGG [7, 8], we treat MT as interval-censored between two successive scans [9] and re-evaluate the preoperative imaging phenotype and the extent of resection within that framework, cross-checking each effect across complementary survival models.

## Methods

### Cohort and endpoint

Eligible patients had a histologically confirmed adult-type diffuse glioma of WHO grade 2 or 3, primary diagnosis and management at our institution, diagnostic-quality preoperative multiparametric MRI (T1, T1c, T2, FLAIR) and at least one subsequent surveillance MRI. Patient selection is shown in Supplementary Figure S4. “Adult-type” denotes the histomolecular entity, not patient age. IDH status was determinable in 151/155; the four indeterminate cases were excluded from IDH-stratified analyses only. All 155 entered the primary analysis, with an age-restricted sensitivity analysis (≥ 18 years, *n* = 149, 76 events; Table S3).

The time origin throughout is the date of the first MRI showing a diffuse glioma, that is, the radiological diagnosis. Histological confirmation, required for eligibility, was obtained at the first operation; in 18 patients (11.6%) this followed the radiological diagnosis by more than one year (median 5.0 years, longest 17.3) under a deliberate wait-and-see strategy, contributing their untreated radiological course to the analysis.

Every included tumour was classifiable as LGG without radiological criteria for malignancy at diagnosis; baseline “contrast enhancement” (CE) therefore denotes sub-threshold enhancement in a tumour read as low-grade. Grading followed WHO 2016, in force at diagnosis; tumours that the 2021 scheme [4, 5] would reassign were retained deliberately, and the effect of hypothetical reclassification is quantified in the Results and in Tables S6 and S13.

MT was ascertained by one of two prespecified routes: histopathological — an unequivocal increase in WHO grade established by a senior neuropathologist on repeat biopsy, resection or autopsy — or radiological — a new, measurable, nodular region of enhancement (≥ 10 mm) by RANO LGG criteria, persisting on the next MRI and a significant change from baseline [3, 19]. Candidate radiological events were adjudicated by a senior attending and a senior neuroradiologist, with a third senior physician as deciding vote, against four criteria: enhancement within 72 h of surgery or confined to the resection margin was attributed to surgical change; enhancement within 12 weeks of radiotherapy inside the high-dose volume was accepted only on persistence with continued growth, or on perfusion or histological corroboration; enhancement in an arterial territory with restricted diffusion was attributed to ischaemia; and enhancement without a corresponding T2/FLAIR mass was not counted.

Each event time was interval-censored by [L, R]. L is the last time point at which low-grade status was documented — the diagnostic MRI for patients transforming before their first operation, and the first operation itself, at which low-grade histology was established, for those transforming afterwards. R is the first examination showing transformation, or the transformation surgery, whichever came first. Because L is fixed to these landmarks rather than to the most recent negative surveillance study, the bracket cannot be narrowed by denser imaging, making the encoding conservative with respect to differential surveillance at the cost of width. Non-transformers were right-censored at their last study; death without MT (*n* = 6) was the competing event (Table S11).

### Imaging analysis

Tumours were segmented on FLAIR, or T2 where FLAIR was unavailable, with the deep-learning-assisted pipeline previously established [9], every segmentation reviewed by neuroradiology. The whole-tumour mask was resampled onto the co-registered T1, T1c and T2 volumes, and PyRadiomics v3.0.1 applied to each after intensity normalisation and 1-mm isotropic resampling (428 features), reduced to 99 by Pearson-correlation filtering [13, 16, 20]. No separate mask of the enhancing sub-compartment was generated.

Baseline CE was scored visually on the post-contrast T1 series against the co-registered pre-contrast T1 at identical window settings. A tumour was CE-positive when both held: (i) at least one focus of unequivocal post-contrast signal increase, visible on two or more contiguous slices; and (ii) a pattern that was neither ring-like nor solid-nodular, without central necrosis, discrete enhancing nodule or multifocal enhancing deposits. A tumour meeting (i) but not (ii) met a radiological criterion for malignancy and was not eligible. The read was morphological, not volumetric (rationale in Supplementary S7). Baseline examinations were re-scored independently by two reviewers with four and five years of experience, not involved in the original assessment and blinded to follow-up, to outcome and to one another; agreement is reported in the Results.

### Statistical analysis

The primary model was a Bayesian interval-censored Weibull with a regularised horseshoe prior [21] over all 102 coefficients (three clinical predictors, 99 radiomic features), fitted in brms/cmdstan [22]; full specification and diagnostics are in Supplementary S1.2. Three frequentist models served as cross-checks: a Fine-Gray subdistribution-hazards model [23] with non-MT death as competing event; a Cox counting-process time-varying-covariate (TVC) model carrying treatment state (pre-surgery; post-surgery by extent of resection) with 20-fold Rubin-pooled multiple imputation of the event time [24]; and a native interval-censored Fine-Gray by sieve maximum likelihood (intccr). Prespecified sensitivities are given in Table S3. Reporting follows STROBE and TRIPOD + AI 2024.

Post-surgical transformation was modelled with time from first surgery (patients transforming at or before surgery excluded; *n* = 128, 50 events) using interval-censored Weibull proportional-hazards models (icenReg). Internal validity of the Cox-TVC fit was assessed by Harrell’s bootstrap-optimism procedure (500 resamples) [25]. Three additional analyses were performed: landmarks at 3, 6 and 12 months; Firth penalised-likelihood and exact conditional estimates where a stratum showed quasi-complete separation; and calibration against the non-parametric Turnbull estimator with time-dependent Brier scores (Supplementary S8–S10).

## Results

### Cohort

**Fig. 1:**
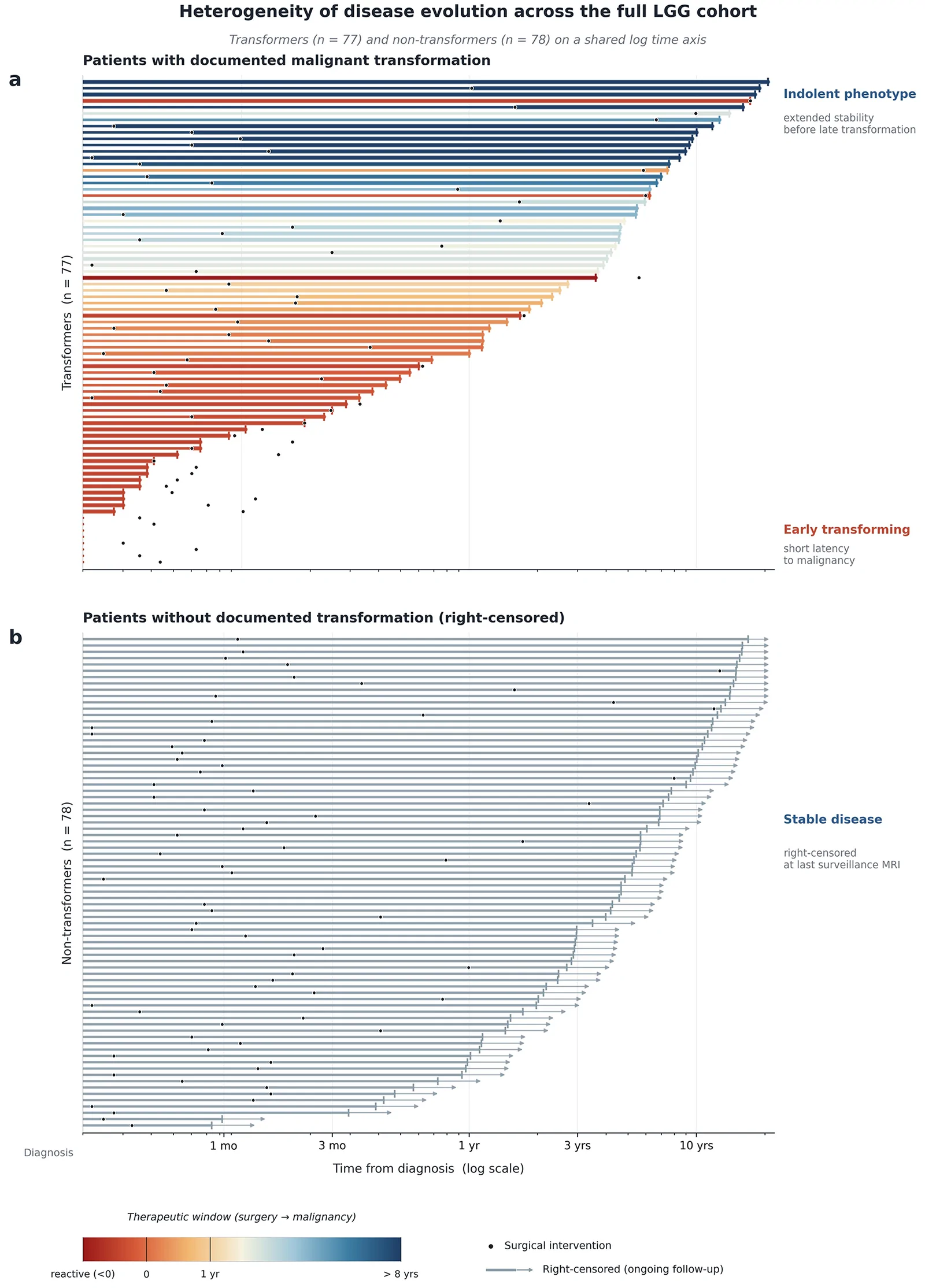
Heterogeneity of disease evolution across the full cohort. Each horizontal bar is one patient; time from diagnosis is on a shared logarithmic axis. (**a**) The 77 patients with documented malignant transformation, ordered by latency from short-latency (“early transforming”) at the bottom to long-latency (“indolent”) at the top; the ordering is a continuum, not discrete subgroups. Bar colour encodes the therapeutic window between surgery and transformation (red = surgery at or after transformation, “reactive”, ≤ 0; blue = surgery > 8 years before transformation). (**b**) The 78 non-transformers, right-censored at last surveillance imaging and ordered by follow-up length; this panel includes the six patients censored at non-MT death. Black dots mark surgical interventions; arrows mark right-censoring. The figure illustrates the interval-censored, competing-risks structure that the analysis is designed to respect

Cohort characteristics by transformation status are summarised in Table 1. Of 155 patients, 77 (49.7%) underwent MT and 78 did not, six of the latter dying without transformation; median surveillance among non-transformers was 4.8 years, 20 (25.6%) beyond ten (Fig. 1). The cohort comprised 116 WHO 2016 grade 2 and 39 grade 3 tumours; reconstructed against the 2021 scheme, 59 IDH-mutant astrocytomas, 47 IDH-mutant 1p/19q-codeleted oligodendrogliomas and 42 IDH-wildtype tumours. Molecular coverage is detailed in Supplementary S8 and Table S12.

**Table 1:** Cohort characteristics by malignant-transformation status (*n* = 155)

Characteristic	All (*n* = 155)	MT (*n* = 77)	No MT (*n* = 78)	*p*
Age at diagnosis, years — median (IQR)	40.1 (30.9–52.0)	43.5 (33.1–52.0)	37.8 (29.8–50.7)	0.107
Female — n (%)	62 (40.0)	30 (39.0)	32 (41.0)	0.922
WHO 2016 grade 3 — n (%)	39 (25.2)	30 (39.0)	9 (11.5)	< 0.001
IDH status — n (%)				0.074
Mutant	109 (70.3)	48 (62.3)	61 (78.2)	
Wildtype	42 (27.1)	26 (33.8)	16 (20.5)	
Indeterminate	4 (2.6)	3 (3.9)	1 (1.3)	
1p/19q status — n (%)				0.064
Codeleted	50 (32.3)	18 (23.4)	32 (41.0)	
Retained	99 (63.9)	53 (68.8)	46 (59.0)	
Not available	6 (3.9)	6 (7.8)	0 (0.0)	
Integrated type (WHO 2021 reconstruction) — n (%)				0.053
Astrocytoma, IDH-mutant	59 (38.1)	28 (36.4)	31 (39.7)	
Oligodendroglioma, IDH-mutant 1p/19q-codeleted	47 (30.3)	17 (22.1)	30 (38.5)	
IDH-wildtype	42 (27.1)	26 (33.8)	16 (20.5)	
Not classifiable	7 (4.5)	6 (7.8)	1 (1.3)	
WHO 2021 molecular grade-4 criterion met — n (%)	21 (13.5)	15 (19.5)	6 (7.7)	0.056
Contrast enhancement at diagnosis (sub-threshold) — n (%)	34 (21.9)	33 (42.9)	1 (1.3)	< 0.001
Tumour volume at diagnosis, cm³ — median (IQR)	35.6 (18.4–66.2)	39.3 (18.7–73.4)	34.2 (16.7–56.1)	0.152
Meso-/archicortical phenotype — n (%)	39 (25.2)	23 (29.9)	16 (20.5)	0.247
Extent of resection — n (%)				0.026
Gross total (GTR)	84 (54.2)	38 (49.4)	46 (59.0)	
Subtotal (STR)	47 (30.3)	21 (27.3)	26 (33.3)	
Biopsy	24 (15.5)	18 (23.4)	6 (7.7)	
Time from diagnosis to first surgery, years — median (IQR)	0.08 (0.04–0.17)	0.07 (0.04–0.19)	0.08 (0.05–0.17)	0.739
First surgery > 1 year after diagnosis — n (%)	18 (11.6)	11 (14.3)	7 (9.0)	0.435
Preoperative MRI examinations, n — median (IQR)	3 (2–3)	3 (2–3)	2 (2–3)	0.606
Chemotherapy, ever — n (%)	95 (61.3)	65 (84.4)	30 (38.5)	< 0.001
Radiotherapy, ever — n (%)	102 (65.8)	65 (84.4)	37 (47.4)	< 0.001
Diagnosis 1998–2009 — n (%)	53 (34.2)	35 (45.5)	18 (23.1)	0.006
Death without transformation — n (%)	6 (3.9)	—	6 (7.7)	—
Surveillance in non-transformers, years — median (IQR)	—	—	4.8 (2.0–10.0)	—
Followed ≥ 10 years — n (%)	—	—	20 (25.6)	—

Consecutive patients with radiologically low-grade glioma diagnosed between 1998 and 2021 at the Department of Neurosurgery, Kepler University Hospital, Johannes Kepler University Linz, Austria, and followed to March 2022. Grading per WHO 2016, in force at diagnosis; the integrated type is a reconstruction against the 2021 scheme from documented IDH and 1p/19q status. “Contrast enhancement” at diagnosis denotes sub-threshold enhancement (minimal or patchy, non-ring-like, non-nodular) in a tumour radiologically classifiable as low-grade. Continuous variables are compared by Mann–Whitney U test and categorical variables by χ², Yates-corrected for 2 × 2 tables. Molecular comparisons are restricted to patients in whom the marker was determined (IDH *n* = 151, 1p/19q *n* = 149, integrated type *n* = 148), so that the test addresses the biological contrast rather than the pattern of missing data. p values are descriptive and unadjusted for multiplicity. Surveillance percentages are of the 78 non-transformers. Complete molecular distributions, including TERT-promoter and CDKN2A/B testing coverage, are given in Supplementary Table S12. IQR, interquartile range

### Preoperative contrast enhancement

**Fig. 2:**
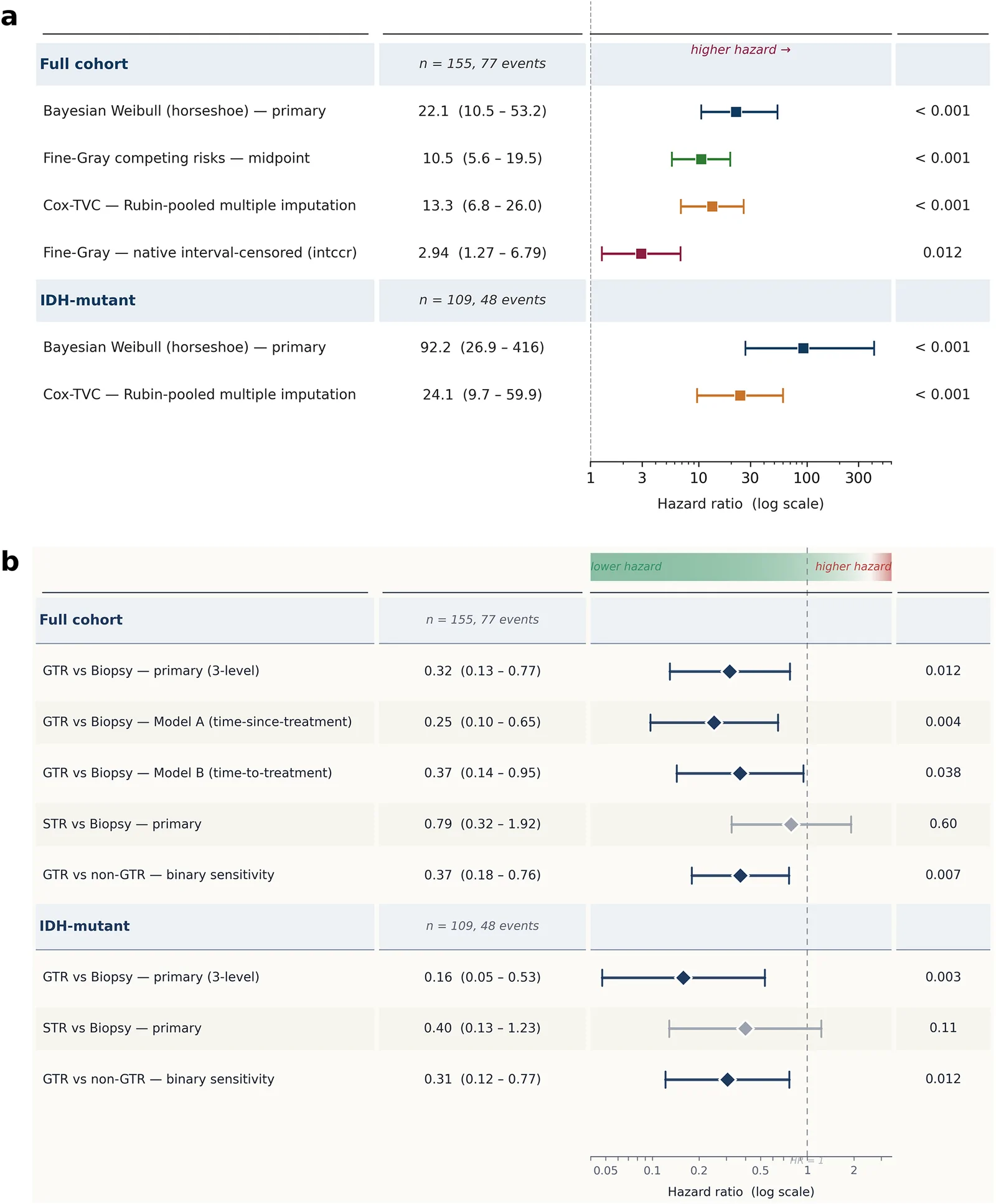
Preoperative contrast enhancement dominates transformation risk, and gross-total resection reduces post-surgical hazard. (**a**) Hazard ratios for the baseline predictors on a log₁₀ scale, across four complementary survival models and three cohorts. The Bayesian interval-censored Weibull is the primary specification (points, posterior medians; whiskers, 95% credible intervals); the three frequentist cross-checks — midpoint Fine-Gray; Cox counting-process model with 20-fold Rubin-pooled multiple imputation of the interval-censored event time; and native interval-censored Fine-Gray by sieve maximum likelihood (intccr) — are shown as point estimates with 95% confidence intervals. Each model addresses a different structural feature of the data (interval censoring; the competing risk of non-MT death; time-varying treatment), and their agreement on direction and significance is the study’s central robustness argument. (**b**) Extent-of-resection contrasts from the Cox counting-process time-varying-covariate model, pooled across 20 imputations by Rubin’s rules; contrasts are versus biopsy unless labelled otherwise, and the prespecified binary sensitivity (†) collapses subtotal resection and biopsy into a single non-GTR reference. Full model output is given in Supplementary Tables S2 and S9 (Methods S1.4–S1.5)

CE at diagnosis dominated transformation risk in every cohort (Fig. 2a; Table S8). In IDH-mutant disease all 22 enhancing tumours transformed versus 30% (26/87) of non-enhancing tumours; with the enhancing/non-transforming cell empty this stratum shows quasi-complete separation, and the Bayesian hazard ratio (92.2, 95% CrI 26.9–416) is a horseshoe-regularised summary whose lower bound rules out a null effect but whose point estimate is not quantitatively interpretable (Firth odds ratio 104, 95% CI 13.6–13446; Tables S6b, S7). The full-cohort Bayesian HR was 22.1 (95% CrI 10.5–53.2), stable across the horseshoe-width grid, with no radiomic feature actively selected. In IDH-wildtype, CE reached HR 10.2 (0.99–168) at *n* = 42. Age and baseline volume were null throughout [26].

Within each represented entity every enhancing tumour transformed (11/11 astrocytoma, 9/9 oligodendroglioma, 11/11 IDH-wildtype) against 17/48, 8/38 and 15/31 of non-enhancing tumours. Because the enhancing, non-transforming cell is empty throughout, stratum-specific effects are reported as Firth penalised and exact conditional estimates, whose lower bounds exclude unity throughout (Table S13). Entered jointly, enhancement remained dominant, grade 3 was independently prognostic (HR 3.06, 1.83–5.08) and IDH-mutant status carried no independent effect (HR 1.01, 0.59–1.73). A meso-/archicortical phenotype [27] was not prognostic (HR 1.51, 0.90–2.52), and the effect persisted in grade-2 tumours alone (18/19 vs. 29/97; Firth OR 28.6, 6.8–267).

Twenty-one patients met a documented WHO 2021 criterion for grade 4 (19 IDH-wildtype with TERT-promoter mutation; two IDH-mutant with CDKN2A/B alteration). Excluding them (*n* = 134, 62 events) left the effect unchanged — 28 of 29 enhancing tumours transformed against 34 of 105 non-enhancing, the hazard ratio moving by + 8% — as did the stricter exclusion of the 22 IDH-wildtype patients without TERT testing (*n* = 112, 50 events). Because EGFR amplification and + 7/−10 were not assessed, these 21 patients are a lower bound on the number the 2021 scheme would reclassify.

Because much of the full-cohort effect reflects tumours already transforming at diagnosis, a prespecified landmark series conditions on transformation-free survival and restarts the clock there. The CE hazard ratio was 7.41 (95% CI 3.26–16.85) at three months (*n* = 128, 52 events), 5.65 (2.23–14.31) at six months (*n* = 119, 47 events) and 6.33 (2.47–16.19) at twelve months (*n* = 111, 44 events); Turnbull cumulative-incidence curves are shown in Figure S5. An unmeasured confounder would need a risk ratio of at least 5.8 with both enhancement and transformation to explain the six-month estimate away (Supplementary S9).

### Radiomic features

Across 99 candidates, no feature’s 95% credible interval excluded the null in the full cohort or the IDH-mutant subgroup (Fig. S3; Table S4). The two features carried forward from prior stability selection sat near HR = 1; one crossed the null in midpoint Fine-Gray (GLDM-FLAIR sHR 1.54, 1.13–2.10), but native interval-censored Fine-Gray collapsed both (Table S1c), so the signature survives only when the event time is compressed to a point [28–31]. This is a conditional null — no reproducibility filter, no harmonisation across 19 scanner models, 77 events for 99 candidates — that bounds what this cohort can show rather than showing that no such signal exists. It is not an artefact of shared measurement: enhancement was scored visually, outside the radiomics pipeline.

### Model concordance and extent of resection

All four models agreed on the direction and significance of the CE effect (midpoint Fine-Gray sHR 10.5, 5.6–19.5; Cox-TVC HR 13.3, 6.8–26.0; native interval-censored Fine-Gray sHR 2.94, 1.27–6.79; all *p* < 0.02); Gray’s test confirmed the effect acts on MT specifically, not through the competing risk of death. GTR markedly reduced post-surgical hazard (Fig. 2b; Table S9): Cox-TVC GTR-versus-biopsy HR 0.32 (0.13–0.77), *p* = 0.012 in the full cohort and 0.16 (0.05–0.53) in IDH-mutant disease [32–34]. STR did not differ from biopsy (HR 0.79, 0.32–1.92).

### Enhancement predicts post-surgical transformation

**Fig. 3:**
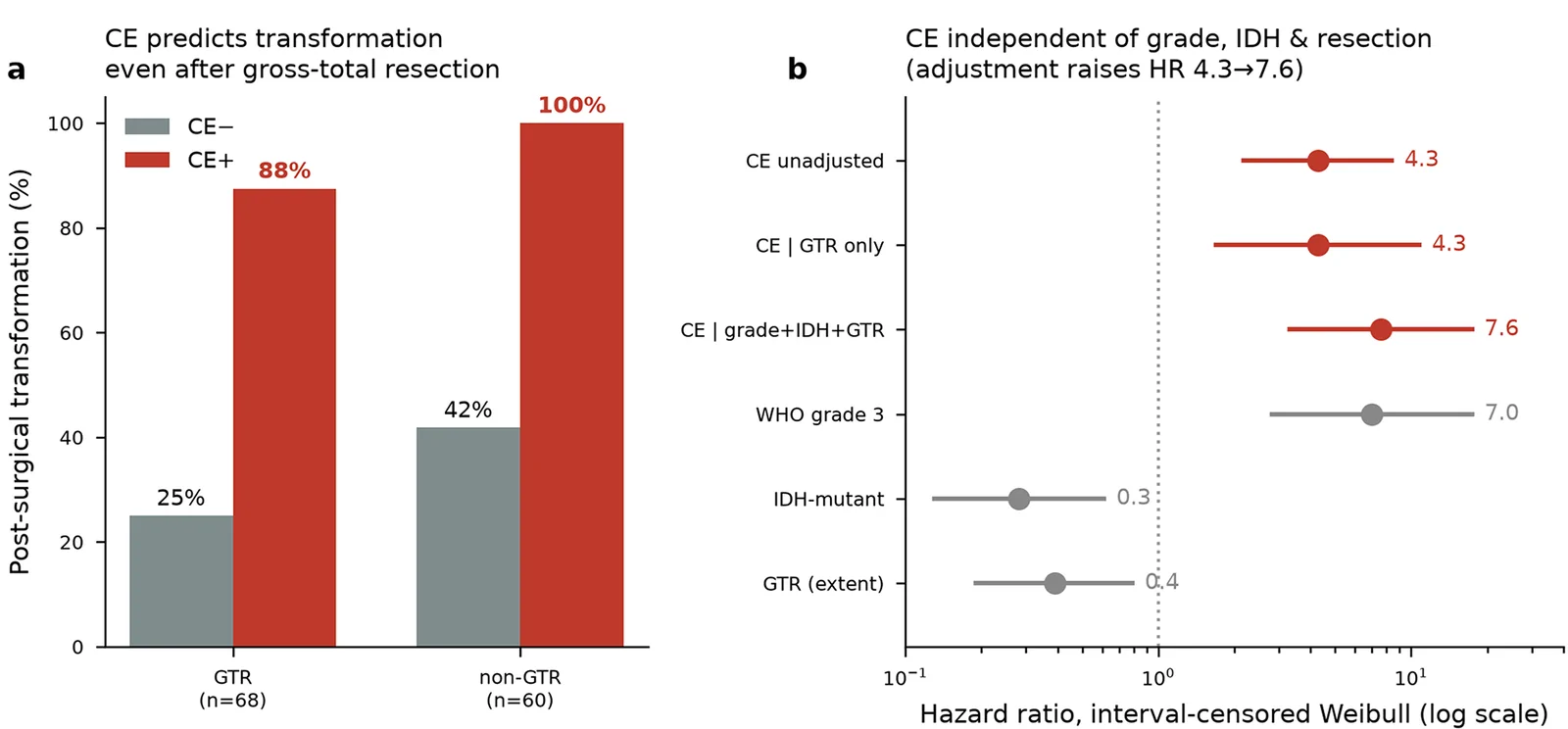
Baseline contrast enhancement predicts post-surgical malignant transformation independently of grade, IDH status and extent of resection. (**a**) Post-surgical transformation rate by baseline enhancement, within gross-total-resection (GTR) and non-GTR strata; enhancing tumours transform more often even after GTR. (**b**) Hazard ratios from interval-censored Weibull proportional-hazards models of time from first surgery (log scale): the enhancement effect is present unadjusted, persists within GTR, and strengthens under adjustment for grade, IDH status and resection extent (4.3 → 7.6), each of which is independently prognostic. Points are hazard-ratio estimates; whiskers are 95% confidence intervals

In an interval-censored Weibull model of time from surgery (*n* = 128, 50 post-surgical transformations), CE at diagnosis predicted transformation with HR 4.28 (95% CI 2.18–8.40). The effect persisted within gross-totally resected tumours (HR 4.28, 1.69–10.79; 7 of 8 CE-positive versus 15 of 60 CE-negative tumours transformed), in which any transforming focus present at baseline should have been removed. Adjustment strengthened rather than attenuated it: with grade, IDH status and resection extent added, each term was independently prognostic (CE HR 7.58, 3.30–17.40; grade 3 h 7.00, 2.81–17.43; IDH-mutant HR 0.28, 0.13–0.61; GTR HR 0.39, 0.19–0.79; all *p* < 0.01; Fig. 3, Table S10).

### Endpoint sensitivity, observation process and internal validation

Endpoint and exposure are related imaging constructs, and event timing differed sharply: 21 of the 33 transforming CE-positive tumours reached the endpoint before the first operation, a median of 11 days after diagnosis (IQR 6–15), whereas CE-negative tumours transformed a median of 4.6 years after diagnosis. Restricting transformation to histologically confirmed events (31 of 77; 30 analysable) attenuated the association in the full cohort (HR 2.2, 95% CI 1.02–4.65, *p* = 0.046) and removed significance in the IDH-mutant subgroup (HR 1.9, 0.73–4.97, *p* = 0.19). With the landmark series, this shows that the unconditioned estimate should not be read as a forecast of distant transformation; the conditioned estimate is the defensible measure of predictive value. Histological confirmation was most frequent among tumours transforming after more than one year (4/7), indicating that sub-threshold enhancement can precede a genuine histological event.

The two blinded reviewers agreed on baseline enhancement in all 46 re-scored cohort examinations (κ = 1.00; κ = 0.98 across all 85 re-scored), and on new enhancement at transformation in 95.7% (κ = 0.91)(full agreement in Table S14). Bracket widths, imaging intensity and time to first operation by exposure, molecular status and era are reported in Supplementary S10 and Table S15: pre-treatment MRI counts were identical in enhancing and non-enhancing patients (median 3, *p* = 0.61), and rounding all brackets onto a common 6- or 12-month grid reduced the CE hazard ratio by 17% and 28%, the confidence interval excluding unity throughout.

**Fig. 4:**
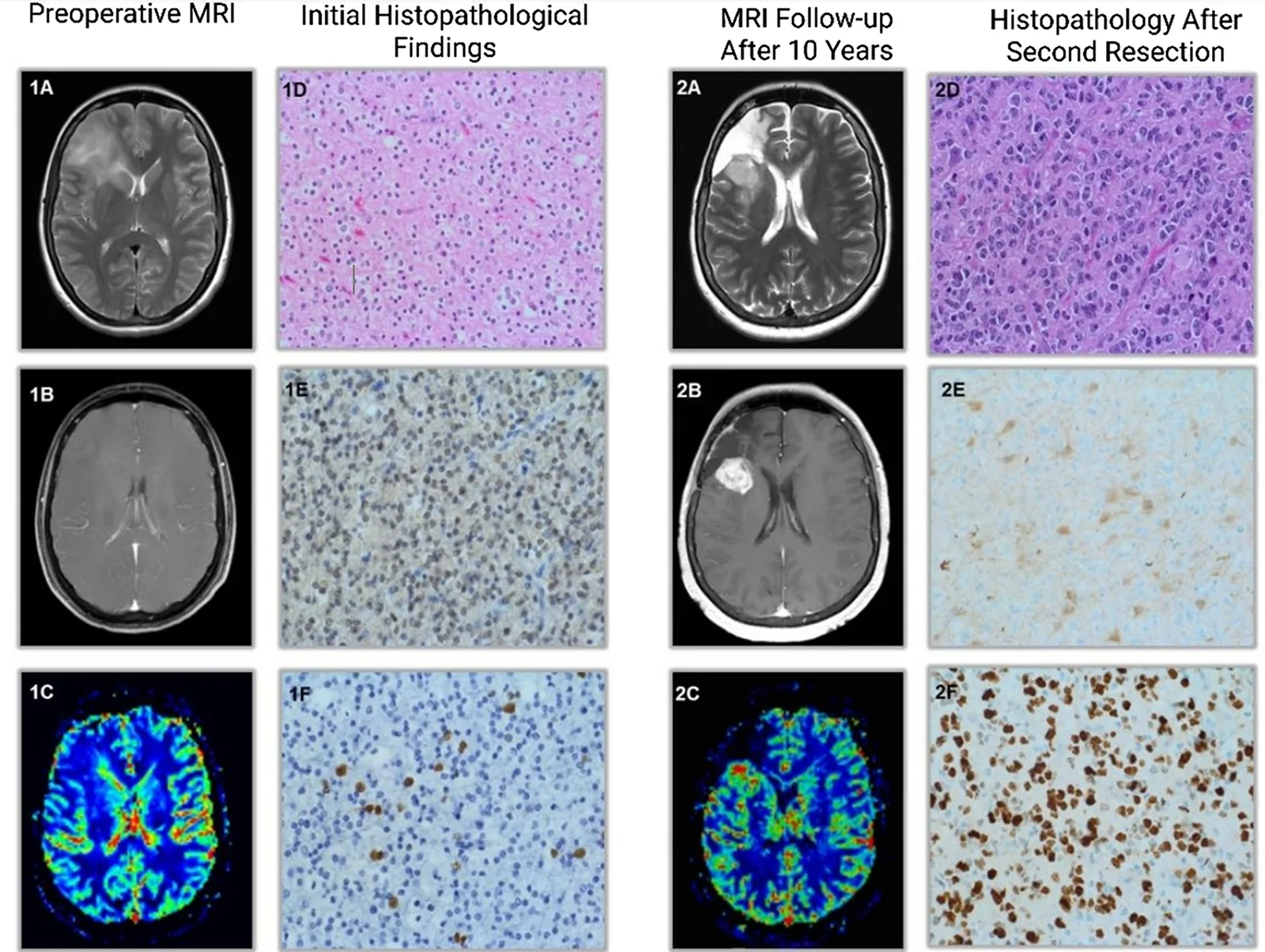
Paired longitudinal imaging and histology in an index oligodendroglioma confirm that the radiological endpoint captures a genuine biological phase transition. WHO grade 2, IDH1-mutant, 1p/19q-codeleted oligodendroglioma imaged across a ten-year interval. Baseline (panels **1A**–**1F**): T2 hyperintensity without measurable post-contrast enhancement on T1c, low relative cerebral blood volume on perfusion MRI; histopathology shows IDH1 R132H-mutated tumour cells with Ki-67 ≈ 7%. Ten-year follow-up (panels 2 A–2 F): new nodular contrast enhancement, elevated rCBV, retained IDH1 R132H clonal identity, anaplastic cytoarchitecture, and Ki-67 ≈ 90%

Bootstrap-optimism validation of the Cox-TVC fit (500 resamples) gave a corrected Harrell C of 0.83, the CE hazard ratio attenuating from 12.1 to a bias-corrected 8.5 (Table S5). Calibration was weaker: the corrected slope was 0.92 (0.67–1.20), but against the non-parametric Turnbull estimator the model over-predicted absolute risk in non-enhancing tumours (five-year probability 0.55 predicted vs. 0.27 observed in CE-negative grade 2 disease), and the time-dependent Brier score improved only modestly on the marginal reference (Table S16). The model discriminates adequately but is not calibrated for individual absolute-risk prediction, and we do not propose it as such. Figure 4 documents an index case of the biological transition the endpoint captures [8, 35–37].

## Discussion

In radiologically low-grade glioma, contrast enhancement at diagnosis was strongly associated with malignant transformation, exceeding tumour volume and a 99-feature radiomic panel. In most enhancing tumours transformation was documented within weeks; conditioned on transformation-free survival to six months, a five-fold hazard remains. Its defining feature is persistence: enhancement measured on the diagnostic scan still predicted transformation after gross-total resection had removed the enhancing tissue, and after adjustment for grade, IDH status and resection extent. A signal that outlives the tissue generating it points to a property of the tumour as a whole rather than a focus awaiting excision. The effect was present in each of the three integrated tumour types.

Agreement across four complementary models makes the effects credible beyond any single specification [11, 23], since each handles a different structural feature of these data under different assumptions. The native interval-censored estimate is about four-fold smaller than the midpoint and multiple-imputation estimates, but direction and significance survive that calibration. Right-censored Cox models equate radiological detection with transformation, incurring bias that grows with the surveillance interval [12, 17].

### Clinical implications

A transformation-risk estimate serves to adjust surveillance intervals and flag tumours needing closer monitoring, not to delay surgery: early onco-functional resection remains the standard of care [38], and our GTR result reinforces it. Because baseline enhancement predicted transformation after surgery independently of grade, IDH status and resection extent, it should be carried into postoperative risk estimation rather than discarded once histology is available. Whether shorter intervals or earlier resection improve outcome in enhancing tumours is a hypothesis these data generate, not a finding they establish, since the decision to operate was frequently prompted by early signs of transformation. At 77 events a 99-feature panel cannot deliver credible individual biomarkers; discovery should be re-scoped to predefined panels or larger cohorts [15, 28].

### Limitations

This is a single-institution, retrospective cohort, and external validation remains the appropriate test of generalisation — harder to assemble here than for most prognostic questions, since resolving the natural history of transformation requires unusually long surveillance. Quantitative residual tumour volume was unavailable, precluding a within-STR analysis.

Exposure and endpoint are both read from post-contrast T1 imaging, and the design limits but cannot eliminate the risk of incorporation bias: eligibility required a sub-threshold baseline and the endpoint a persisting nodular lesion. The unconditioned estimate marks disease already in transition rather than forecasting it; conditioned on transformation-free survival, a five- to seven-fold hazard persists at all three landmarks. The cohort pools biologically distinct entities, and adjustment for IDH status does not substitute for an integrated diagnosis; the analysis was therefore repeated within each integrated type, where the direction held but subgroup size and quasi-complete separation limit precision. Imaging intensity did not differ by enhancement status, but enhancing patients were operated on ten days sooner and only one remained transformation-free, so that group’s surveillance experience is sparsely observed.

Several established predictors could not be evaluated. The velocity of diametric expansion, repeatedly associated with malignant progression [17], requires at least two comparable pre-treatment volumetric examinations; only 18 patients here had more than a year of pre-surgical observation. None of those 18 was CE-positive, so the enhancement effect does not arise within that subgroup, but we cannot say whether enhancement adds information beyond growth kinetics. Seizure at presentation was not recorded in analysable form, and perfusion imaging was obtained non-randomly. The timing of surgery is confounded by indication and immortal-time selection and not causally estimable.

## Conclusion

Preoperative contrast enhancement is the strongest predictor of malignant transformation in lower-grade glioma and identifies a subgroup at markedly elevated risk, for whom shorter surveillance intervals and earlier surgery warrant prospective evaluation. Most enhancing tumours transformed within weeks, but a five- to seven-fold hazard persists after conditioning on transformation-free survival. Four complementary survival models agree on its direction and significance; gross-total resection produces a large postoperative hazard reduction; and no radiomic feature survives Bayesian shrinkage or native interval-censored analysis at this cohort size. The prognostic value of enhancement is not exhausted at surgery: it predicts post-surgical transformation independently of grade, IDH status and resection extent — after gross-total resection has removed the enhancing tissue — and belongs in postoperative as well as preoperative risk estimation.

## Supplementary Information

Below is the link to the electronic supplementary material.

Supplementary Material 1

## Data Availability

The de-identified radiomic feature matrix is available from the corresponding author on reasonable request. Individual-level clinical and imaging data cannot be publicly deposited owing to data-protection conditions imposed by the JKU Ethics Committee, but are available from the corresponding author on reasonable request, subject to institutional and ethical approval.

## Code availability

All analysis code is publicly available at: https://github.com/PRauch1/Malignant-transformation-in-lower-grade-glioma--a-Bayesian-interval-censored-analysis- Full numerical outputs, Supplementary Tables S1a–S16 and Supplementary Figures S1–S5 accompany this manuscript.

## Declarations

### Use of generative AI

Generative-AI technologies were used during manuscript preparation solely to improve clarity and readability of the written text and to edit and format tables and figures. All AI output was reviewed and revised by the authors, who accept full responsibility for the final content.
