# Supplementary material for: Malignant transformation of lower-grade glioma: contrast enhancement, extent of resection, and the natural history under interval-censored analysis
Source: J Neurooncol. 2026 Sep 22;179(3):94. doi: 10.1007/s11060-026-05803-0 (PMC13597587; doi:10.1007/s11060-026-05803-0)
Supplement: Supplementary file 1 [file 11060_2026_5803_MOESM1_ESM.docx]

**Supplementary Materials**

*Malignant transformation of lower-grade glioma: contrast enhancement, extent of resection, and the natural history under interval-censored analysis*

**Journal of Neuro-Oncology — Supplementary Information (Online Resource)**

Baran Atli, Maximilian Wartha, Matthias Gmeiner, Martin Aichholzer, Serge Weis, Dominik Vorhauer, Helga Wagner, Tanja Hauer, Carlo Serra, Petra Böhm, Michael Sonnberger, Nico Stroh-Holly, Stefan Aspalter, Kathrin Aufschnaiter, Tobias Rossmann, Maria Gollwitzer, Jawed Ziai, Annette Leibetseder, Josef Pichler, Wolfgang Thomae, Christian Dorfer, Andreas Gruber, Harald Stefanits, Philip Rauch

Corresponding author: Maximilian Wartha, MD — Department of Neurosurgery, Kepler University Hospital, Neuromed Campus, Wagner-Jauregg-Weg 15, 4020 Linz, Austria — Maximilian.Wartha@kepleruniklinikum.at

# S1. Extended methods

## S1.1 Interval-censored event-time encoding

For each patient the MT event time was encoded as an interval [L, R], where L is the date of the last pre-MT imaging study classified as low-grade and R is the date of the first imaging study showing MT (or the diagnostic surgery at transformation, whichever was earlier). Patients without documented transformation were right-censored at the last available imaging follow-up (L = last imaging date, R = ∞). Patients who underwent surgery before any observed MT were additionally stratified into Case 2 (MT observed in [L, R] prior to surgery) and Case 3 (MT observed in [L, R] after surgery); Supplementary Figure S1 shows the schematic. All three Bayesian, Fine-Gray, and Cox-TVC analyses were specified to respect this interval-censored structure.

**Supplementary Figure S1 | Interval-censored event-time structure: every transformation time is localised to an interval between two successive imaging studies.** Three illustrative cases. **Case 1** — right-censored at the last transformation-free imaging study, with no upper bound on the true MT time. **Case 2** — transformation is detected on the first post-operative MRI, producing an interval [L, R] bounded below by the pre-operative scan and above by the post-operative scan. **Case 3** — transformation is detected after a period of stable post-operative surveillance, producing an interval bounded below by the last stable MRI and above by the first MRI showing transformation. Cases 2 and 3 differ only in whether surgery precedes or follows the lower interval bound L.

## S1.2 Primary Bayesian analysis — full specification

brms 2.23 formula: y | cens(...) ~ contrast_enhancing + age_atDiag45 + vol_diag_centered + {99 radiomic features}. brms parameterises Weibull as accelerated-failure-time (AFT) with a log link on the scale μ; the proportional-hazards HR for covariate j is HR = exp(−α·βj), with α the Weibull shape. HRs and 95% credible intervals were computed draw-by-draw from joint α/β posterior draws, preserving joint uncertainty. A horseshoe prior was placed on all 102 population-level coefficients (par_ratio = 0.1, df = 1, df_global = 1, scale_global = 2, scale_slab = 2, df_slab = 4); the wide slab preserves strong clinical effects while heavily shrinking weak signals. The horseshoe was applied to all coefficients (clinical and radiomic) because brms 2.23 does not permit mixing class-wide horseshoe with per-coefficient priors on the same b class.

Sampling used 4 chains × 8 000 iterations × 4 000 warmup with adapt_delta = 0.999 and max_treedepth = 14 after a documented retry ladder. Divergent-transition counts were 17/16 000 (0.11%) in the full cohort, 11/16 000 (0.07%) in IDH-mutant, and 67/16 000 (0.42%) in IDH-wildtype, all below conventional thresholds and — as documented by a per-parameter divergence-location diagnostic — concentrated in horseshoe auxiliary scale hyperparameters (maximum |z-score| = 2.88 on a local-shrinkage scale) rather than in population-level coefficients of interest (|z-score| ≤ 0.28 for all four clinical coefficients). R-hat ≤ 1.0014 and minimum ESS_bulk ≥ 1 839 in every cohort. A prior-sensitivity refit at scale_global = 1 produced clinical HRs identical to three significant figures.

WHO grade and IDH status were not added to the horseshoe model. The regularised horseshoe applies a common shrinkage prior to all population-level coefficients; placing two strong, well-established clinical predictors under the same sparsity-inducing prior as 99 exploratory texture features would shrink them towards a null known a priori to be false and distort the multiplicity adjustment the prior is intended to provide. The principled alternative is a partially pooled specification exempting grade and IDH from shrinkage, which brms 2.23 does not support within a single coefficient class. The primary model was therefore not refitted in that form: the frequentist multivariable model reported in the main text, with enhancement, WHO grade and IDH status entered jointly, addresses the same question without that specification risk, and the IDH-stratified fits condition on IDH status exactly.

## S1.3 Fine-Gray competing-risks analysis

A Fine-Gray subdistribution-hazards model (R package cmprsk) treated MT as the primary event (n = 77) and death without MT as competing (n = 6). Two specifications were run: clinical-only (contrast enhancement, centred age, centred volume) and clinical + two-feature signature (GLDM-FLAIR dependence-variance and GLSZM-T1c large-area-low-gray-level-emphasis). Gray's test was computed for cumulative-incidence differences by CE strata for both events to rule out competing-risk confounding of the CE signal. The primary cmprsk implementation uses the interval midpoint (L + R) / 2 as the transformation event time. Two additional methodological refinements were specified alongside the primary analysis: (i) multiple imputation of the event time from Uniform(L, R) with M = 20, pooled by Rubin’s rules (Table S1b); and (ii) a native interval-censored Fine-Gray fit via sieve maximum likelihood with B-spline approximation of the cumulative incidence functions (R package intccr, Park & Bakoyannis 2021), integrating the subdistribution-hazards likelihood directly over the bracket [L, R] (Table S1c).

### Supplementary Table S1a. Fine-Gray subdistribution hazards — midpoint plug-in (clinical + 2-feature signature)

| **Term** | **sHR (95% CI)** | **p-value** | **Interpretation** |
| --- | --- | --- | --- |
| Contrast enhancement | 9.87 (5.56, 17.5) | < 0.0001 | strong ↑ |
| Age (per year above 45) | 1.01 (0.99, 1.03) | 0.32 | null |
| Volume (per cm³) | 1.00 (0.99, 1.01) | 0.84 | null |
| GLSZM-T1c large-area-low-gray-level | 1.17 (0.99, 1.37) | 0.064 | borderline |
| GLDM-FLAIR dependence-variance | 1.54 (1.13, 2.10) | 0.006 | credible ↑ |

*Gray’s test* for cumulative incidence by CE strata: MT statistic 80.0, p < 0.0001; death without MT statistic 1.61, p = 0.205. The CE signal is therefore concentrated in the primary event and cannot be explained by a competing-risk mortality process.

### Supplementary Table S1b. Fine-Gray subdistribution hazards — multiple imputation (M = 20, Rubin-pooled)

| **Term** | **sHR (95% CI)** | **p-value** | **Interpretation** |
| --- | --- | --- | --- |
| Contrast enhancement | 9.81 (5.15, 18.7) | < 0.0001 | strong ↑ |
| Age (per year above 45) | 1.01 (0.99, 1.03) | 0.30 | null |
| Volume (per cm³) | 1.00 (0.99, 1.01) | 0.78 | null |
| GLSZM-T1c large-area-low-gray-level | 1.15 (0.94, 1.40) | 0.18 | null |
| GLDM-FLAIR dependence-variance | 1.50 (1.09, 2.05) | 0.012 | credible ↑ |

Multiple imputation seeds are matched to the Cox TVC analysis (2026 + m) so identical event-time draws appear in both pipelines. The midpoint and MI sHRs are essentially identical (CE 9.87 vs 9.81; GLDM-FLAIR 1.54 vs 1.50), indicating that the midpoint plug-in is not a wrong answer but is under-honest about the CI width; multiple imputation is the preferred reporting form.

***Supplementary Table S1c. Native interval-censored Fine-Gray (intccr) — cause 1 = MT, clinical + 2-feature signature***

| **Term** | **sHR (95% CI)** | **p-value** | **Interpretation** |
| --- | --- | --- | --- |
| Contrast enhancement | 2.94 (1.27, 6.79) | 0.012 | credible ↑, attenuated |
| Age (per year above 45) | 1.00 (0.98, 1.02) | 0.92 | null |
| Volume (per cm³) | 1.00 (1.00, 1.01) | 0.81 | null |
| GLSZM-T1c large-area-low-gray-level | 1.05 (0.86, 1.26) | 0.65 | null |
| GLDM-FLAIR dependence-variance | 1.04 (0.81, 1.34) | 0.77 | null |

Fitted by *intccr::ciregic()* with alpha = c(0, 0) (proportional subdistribution hazards for both event types), B-spline sieve approximation of the cumulative incidence functions, and the closed-form least-squares variance estimator of Park & Bakoyannis (2021, §3.4). Cause 2 (death without MT, n = 5 with known time-of-death) is too sparse for stable estimates and is not reported. Under native interval-censoring — in which the subdistribution-hazards likelihood integrates over the full bracket [L, R] rather than reducing it to a representative time — the CE effect attenuates from sHR ~10 (midpoint / MI) to sHR ~2.8 while preserving direction and statistical significance, and both pre-specified signature features collapse to null. This brings the frequentist competing-risks analysis into concordance with the Bayesian horseshoe primary analysis (0 / 99 features credibly non-null) and supports the interpretation that the previously reported “GLDM-FLAIR credibly non-null in unshrunken Fine-Gray” signal was a midpoint-plug-in artifact rather than a population-level effect. We initially tried an illness–death implementation, *SmoothHazard::idm()* (illness-death model with penalized M-splines on cause-specific hazards), but the optimizer did not converge on this dataset under either Weibull or spline baselines; *intccr* is methodologically equivalent for the Fine-Gray (subdistribution-hazards) question and converged cleanly.

## S1.4 Cox counting-process TVC with multiple imputation

Counting-process data were constructed with one row per patient per treatment-state epoch (pre-surgery; post-surgery-by-EOR; post-chemo; post-radiation). Because the event time is interval-censored, 20 imputations of the event time were drawn as Uniform(L, R). The counting-process Cox model was refit in each imputation with coxph including id = patient_number for within-patient robust SE, and log-HR coefficient vectors were pooled via Rubin's rules. Two TVC specifications are reported: a primary spec with step-function treatment indicators and five-level EOR-state factor {pre, GTR, STR, Biopsy, unknown}, and a Model A extension with continuous time-since-treatment covariates. A previously included Model B extension that placed the per-patient time-to-first-surgery as a baseline covariate in all intervals was not retained in the primary specification because the regressor is not measurable at the time the hazard is modeled (anticipation bias); the effect was null (HR 1.03, p = 0.53) and removal does not affect any substantive conclusion. A rank-deficiency between post_surgery and the full EOR factor forces the Biopsy level to be dropped, so the reported GTR and STR coefficients from the primary spec represent GTR-vs-Biopsy and STR-vs-Biopsy contrasts. The pre-specified binary EOR sensitivity (GTR vs non-GTR; STR + Biopsy pooled) was designed to remove this rank-deficiency.

### Supplementary Table S2. Cox counting-process TVC — primary spec (full cohort, Rubin-pooled over 20 imputations)

| **Term** | **HR (95% CI)** | **p-value** | **Interpretation** |
| --- | --- | --- | --- |
| Contrast enhancement | 13.3 (6.83, 26.0) | < 0.0001 | strong ↑ |
| Age (per year above 45) | 1.01 (0.99, 1.03) | 0.33 | null |
| Volume (per cm³) | 1.00 (0.99, 1.01) | 0.87 | null |
| eor_stateGTR vs Biopsy | 0.32 (0.13, 0.77) | 0.012 | protective |
| eor_stateSTR vs Biopsy | 0.79 (0.33, 1.92) | 0.60 | null |
| post_chemo (step-function TVC) | 2.75 (0.95, 8.01) | 0.063 | marginal ↑ (selection) |
| post_radiation (step-function TVC) | 0.63 (0.20, 1.96) | 0.42 | null |

Continuous time-since-treatment terms were not informative: time since surgery HR 0.65 (0.20, 2.15), p = 0.48; time-to-first-surgery as baseline covariate HR 1.03 (0.94, 1.14) per year of delay, p = 0.53. The time-since-radiation pooled coefficient was numerically pathological (driven by a thin-data extreme in at least one imputation) and is reported with explicit uninterpretable annotation; this does not affect any primary conclusion.

## S1.5 Binary extent-of-resection sensitivity

Because subtotal resection spans a heterogeneous population from near-complete resections to near-biopsy resections, and because quantitative residual tumour volume was not available, we pre-specified a binary sensitivity collapsing STR and Biopsy into a single non-GTR category. Using the same counting-process MI structure with post_surgery omitted (redundant with the three-level binary factor), GTR vs non-GTR yielded a significant and strongly protective linear contrast: HR 0.370 (0.180, 0.762), p = 0.007 full cohort; HR 0.306 (0.122, 0.767), p = 0.012 IDH-mutant. The magnitude was slightly attenuated compared with GTR-vs-Biopsy alone (0.37 vs 0.32 full cohort; 0.31 vs 0.16 IDH-mutant), consistent with STR sitting intermediate between GTR and Biopsy on the transformation-risk continuum.

Clinical and chemo/radiation effects were materially unchanged by the EOR reparameterization (CE HR 13.6 vs 13.3; post_chemo HR 2.72 vs 2.75).

# S2. Pre-specified sensitivity analyses

### Supplementary Table S3. Methodology sensitivities on the primary Bayesian model

| **Sensitivity** | **CE HR (95% CrI)** | **Note** |
| --- | --- | --- |
| Primary (par_ratio = 0.10) | 22.1 (10.5, 53.2) | reference |
| Horseshoe par_ratio 0.05 | 21.7 (10.3, 53.1) | 0 active radiomic features |
| Horseshoe par_ratio 0.20 | 22.7 (10.5, 53.1) | 0 active radiomic features |
| Age ≥18 years only (n = 149) | 21.3 (9.9, 53.1) | excludes 6 paediatric-age patients with adult-type tumours |
| Era split (≥2010) | 21.9 (10.3, 53.5) | era HR 1.00 (0.93, 1.05) |

Headline inferences are stable across all three pre-specified methodology sensitivities.

# S3. Radiomic shrinkage — supporting detail

The posterior densities of all 99 candidate features under the regularized-horseshoe prior, together with closer portraits of the two pre-specified signature features, are shown in Supplementary Figure S3. The table below provides numeric detail for the top-ranked features; the unshrunken frequentist Fine-Gray refit of the pre-specified two-feature signature is reported above in Table S1.

### Supplementary Table S4. Top 5 radiomic features by |posterior β| (full cohort)

| **Feature** | **HR (95% CrI)** | **CrI excl. 0** | **Pr(\|HR−1\| ≥ 20%)** |
| --- | --- | --- | --- |
| shape_surfacevolumeratio_T1c | 1.00 (0.62, 1.02) | no | 0.16 |
| shape_flatness_FLAIR | 1.00 (0.98, 1.51) | no | 0.14 |
| gldm_dependencevariance_FLAIR * | 1.00 (0.98, 1.38) | no | 0.06 |
| firstorder_10percentile_FLAIR | 1.00 (0.98, 1.23) | no | 0.03 |
| glszm_largearealowgraylevel_T1c * | 1.00 (0.97, 1.15) | no | 0.02 |

** pre-specified two-feature signature.*

## S3a. Internal validation of the primary Cox-TVC model

### Supplementary Table S5. Bootstrap-optimism correction (Harrell, 500 patient-level resamples)

Harrell bootstrap-optimism correction over 500 patient-level resamples (486 converged) of the primary counting-process Cox model. Because each replicate refits the full TVC model, we used a single deterministic mid-interval imputation of the interval-censored event time per replicate rather than the 20-fold MI pipeline applied to the primary report; the apparent HR from this single-imputation fit (12.1) is therefore slightly lower than the Rubin-pooled primary (13.3, 6.8–26.0). For each replicate we fit the Cox-TVC model on the bootstrap sample (boot), then evaluated its linear predictor on the original 155-patient sample (test); optimism is the mean boot–test gap.

| **Metric** | **Apparent** | **Optimism (mean)** | **Corrected** |
| --- | --- | --- | --- |
| Harrell C (discrimination) | 0.847 | 0.018 | 0.83 |
| CE log-hazard ratio | 2.49 | +0.35 | 2.14 |
| CE hazard ratio | 12.1 | — | 8.5 (bootstrap 95% CI 7.7–57) |

The optimism on discrimination is modest (ΔC ≈ 0.02). The bootstrap coefficient distribution is right-skewed because a minority of resamples draw many CE = Y patients and drive large log-HRs; the bias-corrected point estimate shrinks the apparent HR from 12.1 to 8.5, and the bootstrap 95% percentile interval (7.7–57) is wider than but consistent with the Rubin-pooled primary CI. Internal validation is not a substitute for external validation in an independent cohort, which remains the pre-registered next step.

# S4. Analytic-design overview

**Supplementary Figure S2 | Four complementary survival models applied to the same interval-censored cohort address different structural features of the data (interval-censored event time, competing risk of non-MT death, time-varying treatment exposures); their agreement is the study's central robustness argument. Primary: interval-censored Bayesian Weibull model with a horseshoe prior on all 102 population-level coefficients (par_ratio = 0.1, scale_slab = 2, df_slab = 4). Hazard ratios are computed draw-by-draw from the joint AFT posterior. Secondary: Fine-Gray subdistribution-hazards model treating MT as the primary event and non-MT death as competing, with a pre-specified two-feature radiomic signature evaluated without shrinkage. Secondary: Cox counting-process time-varying-covariate model with 20-fold multiple imputation of the interval-censored event time, pooled across imputations by Rubin’s rules. Secondary: Fine-Gray fit with native interval-censored sieve maximum likelihood and B-spline approximation of the cumulative incidence functions (intccr; Park & Bakoyannis 2021), integrating the subdistribution-hazards likelihood directly over the bracket [L, R] rather than relying on a midpoint or imputation plug-in. Pre-specified methodology sensitivities — horseshoe par_ratio grid {0.05, 0.10, 0.20}, adult-only restriction, era split at diagnosis year 2010 — are run on the primary model.**

**Supplementary Figure S3 |** Under horseshoe shrinkage, every 95 % posterior credible interval among the 99 candidate radiomic features contains the null; the two signature features carried forward from prior internal bootstrap stability selection are also centred near HR = 1. (a) Posterior densities of the per-feature hazard-ratio coefficients for all 99 radiomic candidates in the full-cohort Bayesian interval-censored Weibull model with a horseshoe prior (par_ratio = 0.1); inset labels report each signature feature's posterior median hazard ratio with its 95 % credible interval. No feature has 95 % posterior mass away from HR = 1. (b, c) Posterior density portraits of the two signature features.

# S5. Malignant-transformation definition — supporting analyses

### Supplementary Table S6. Documented molecular markers across the cohort with implications for a hypothetical WHO 2021 reclassification, and robustness of the headline IDH-mutant CE finding.

## S6a. Molecular-marker distribution and event rates

Counts of IDH × TERT-promoter and IDH × CDKN2A/B status across the 155-patient cohort. Event counts (n with documented malignant transformation) are shown in parentheses. WHO 2021-reclassifying status is highlighted in the right-most column.

| **IDH status** | **Marker** | **Marker value** | **n (events)** | **Under WHO 2021** |
| --- | --- | --- | --- | --- |
| IDH-mutant (n = 109) | CDKN2A/B | Yes (altered) | 2 (2) | → grade 4 astrocytoma |
|  |  | No | 58 (30) | remains grade 2/3 |
|  |  | Not tested | 49 (16) | status indeterminate |
| IDH-wildtype (n = 42) | TERT promoter | Mutated | 19 (13) | → glioblastoma |
|  |  | Wildtype / unclear | 1 (1) | remains LGG (per TERT) |
|  |  | Not tested | 22 (12) | status indeterminate |
| IDH-indeterminate (n = 4) | — | Excluded from IDH-subgroup fits | 4 (—) | — |

Counts in the 'Marker value' rows sum to the IDH-stratum total. Of the 42 IDH-wildtype patients, 19 (45 %) carried documented TERT-promoter mutations sufficient on their own — in IDH-wildtype tumours with low-grade morphology — to support WHO 2021 reclassification to glioblastoma; a further 22 had no TERT-promoter testing on record, leaving the actual WHO 2021 reclassification rate of the IDH-wildtype subgroup at ≥ 45 %. Among the 109 IDH-mutant patients, only 2 (1.8 %) had documented CDKN2A/B alterations triggering WHO 2021 grade-4 reassignment.

## S6b. Robustness of the IDH-mutant CE finding to WHO 2021 reclassification

2 × 2 distribution of contrast enhancement at diagnosis against MT in the IDH-mutant subgroup, before and after exclusion of the 2 CDKN2A/B-altered patients (a hypothetical WHO 2021 reclassification). The CE-positive, IDH-mutant stratum is essentially unchanged; every CE-positive patient underwent MT in both versions.

| **Specification** | **CE status** | **MT** | **no MT** |
| --- | --- | --- | --- |
| Primary cohort (n = 109) | CE-positive | 22 | 0 |
|  | CE-negative | 26 | 61 |
| Hypothetical WHO 2021 (n = 107; excludes CDKN2A/B-altered) | CE-positive | 21 | 0 |
|  | CE-negative | 25 | 61 |

The MT rate among IDH-mutant CE-positive patients is 100 % under eiter specification. The headline IDH-mutant Bayesian HR 92.2 (95 % CrI 26.9–416) is therefore not driven by WHO 2021 reclassifying molecular alterations.

## Note on the IDH-wildtype subgroup

Within the 42-patient IDH-wildtype stratum, contrast enhancement at diagnosis predicts transformation regardless of TERT-promoter status. Among the 19 TERT-mutated patients, all 4 CE-positive patients underwent MT (4/4); among the 22 TERT-untested patients, all 6 CE-positive patients underwent MT (6/6). The wide credible interval of the IDH-wildtype CE estimate (Bayesian HR 10.2, 95 % CrI 0.99–168) is therefore an uncertainty driven by sample size and stratum heterogeneity, not by absence of a CE signal in the data. We retain these patients in the primary analysis because the radiological and histopathological natural history of phenotypically low-grade, molecularly aggressive tumours under conservative management had not been systematically described in the LGG era and is no longer observable in contemporary practice, which reclassifies and treats such tumours as glioblastoma at diagnosis. A complete WHO 2021 reclassification of the cohort is not feasible from the available clinical-database fields (chromosome +7/–10 status and EGFR amplification testing are not on record); the ≥ 45 % TERT-mutated fraction is a lower bound.

### Supplementary Table S7. Firth penalised-likelihood logistic regression (odds of ever transforming). Because the IDH-mutant enhancing/non-transforming cell is empty, the ordinary logistic estimate is not identifiable (complete separation; SE ≈ 1.4×10³); Firth penalisation gives a finite estimate. Full cohort OR 38.9 (9.76–354); grade-adjusted 34.4 (8.46–316); IDH-mutant 104 (13.6–13446). This is a separation diagnostic, distinct from the bootstrap-optimism analysis (Table S5) and from the STROBE and TRIPOD+AI checklists supplied as separate files.

| **Cohort** | **Term** | **Odds ratio (95% profile-likelihood CI)** | **p** |
| --- | --- | --- | --- |
| Full cohort | CE (unpenalised logistic) | not estimable (quasi-separation) |  |
| Full cohort | CE (Firth penalised) | 38.9 (9.76–354) | 1.8e-11 |
| Full cohort, grade-adj | CE (Firth penalised) | 34.4 (8.46–316) | 5.8e-10 |
| IDH-mutant | CE (unpenalised logistic) | not estimable (complete separation; SE≈1.4e3) |  |
| IDH-mutant | CE (Firth penalised) | 104 (13.6–13446) | 2.7e-10 |

### Supplementary Table S8. Bayesian primary clinical effects

| **Cohort** | **Parameter** | **HR (95% CrI)** | **Pr(HR > 1)** |
| --- | --- | --- | --- |
| Full (n = 155, 77 events) | Contrast enhancement | 22.1 (10.5, 53.2) | 1.000 |
|  | Age (per year above 45) | 1.01 (1.00, 1.03) | 0.852 |
|  | Volume (per cm³) | 1.00 (1.00, 1.01) | 0.806 |
| IDH-mutant (n = 109, 48 ev.) | Contrast enhancement | 92.2 (26.9, 416) | 1.000 |
| IDH-wildtype (n = 42, 26 ev.) | Contrast enhancement | 10.2 (0.99, 168) | 0.924 |

### Supplementary Table S9. Extent-of-resection and contrast-enhancement effects (Cox counting-process time-varying-covariate model, 20-fold multiple imputation)

| **Contrast** | **Cohort** | **HR (95% CI)** | **p** |
| --- | --- | --- | --- |
| Contrast enhancement | Full | 13.3 (6.83, 26.0) | < 0.0001 |
| GTR vs Biopsy | Full | 0.32 (0.13, 0.77) | 0.012 |
| GTR vs Biopsy | IDH-mutant | 0.16 (0.05, 0.53) | 0.003 |
| STR vs Biopsy | Full | 0.79 (0.33, 1.92) | 0.60 |
| GTR vs non-GTR † | Full | 0.37 (0.18, 0.76) | 0.007 |
| GTR vs non-GTR † | IDH-mutant | 0.31 (0.12, 0.77) | 0.012 |

### Supplementary Table S10. Post-surgical malignant transformation — interval-censored Weibull proportional-hazards models (time from first surgery)

| **Predictor** | **Model / cohort** | **HR (95% CI)** | **p** |
| --- | --- | --- | --- |
| Contrast enhancement | Unadjusted, all post-surgical (n=128, 50 events) | 4.28 (2.18, 8.40) | < 0.001 |
| Contrast enhancement | Gross-total resection only (n=68, 22 events) | 4.28 (1.69, 10.79) | 0.002 |
| Contrast enhancement | Adjusted for grade (n=128) | 3.99 (1.93, 8.27) | < 0.001 |
| Contrast enhancement | Adjusted for IDH status (n=125) | 6.03 (2.98, 12.19) | < 0.001 |
| Contrast enhancement | Full model † | 7.58 (3.30, 17.40) | < 0.001 |
| WHO grade 3 (vs 2) | Full model † | 7.00 (2.81, 17.43) | < 0.001 |
| IDH-mutant (vs wildtype) | Full model † | 0.28 (0.13, 0.61) | 0.001 |
| Gross-total resection | Full model † | 0.39 (0.19, 0.79) | 0.009 |

*Interval-censored Weibull proportional-hazards models (icenReg::ic_par, model = "ph"); event time measured from first surgery, transformation interval bracketed by the last low-grade and first transformed post-surgical scan; non-transformers right-censored at last post-surgical scan. † Full model contains contrast enhancement, WHO 2016 grade, IDH status and extent of resection simultaneously (n = 125, 48 events); each term is independently prognostic. Adjustment raises the enhancement hazard ratio (4.3 → 7.6), consistent with a predictor not confounded by the tissue variables.*

# Supplementary material added in revision

Sections S6–S10, Tables S11–S16 and Figures S4–S5 were added during revision and are numbered in order of first citation in the main text. Each is referenced from the main text at the point at which the corresponding material was moved out of the body.

# S6. Cohort accounting: participant flow and competing events

Of 288 patients in the institutional glioma database with a histologically confirmed diffuse glioma, 133 were excluded and 155 were analysed (Figure S4). Six of the 78 non-transformers died without transformation and are treated as a competing event throughout (Table S11); all six were non-enhancing at diagnosis, so the competing risk is not differentially distributed with respect to the exposure.

**Supplementary Figure S4 | Participant flow.** Of 288 patients in the institutional glioma database with a histologically confirmed diffuse glioma, 133 were excluded and 155 were analysed. Exclusions for a missing Karnofsky score at admission and for rare categories of tumour side, intraparenchymal location and lobe (22 patients in total) are inherited from the original analysis plan, in which those covariates were screened; they are not covariates of the models reported here. Case 1 denotes tumours already meeting radiological criteria for malignancy at the diagnostic examination, which are outside the scope of this study.

**Supplementary Table S11.** The six patients with a competing event (death without malignant transformation). All six were non-enhancing at diagnosis. Gray's test for the cumulative incidence of death without transformation by CE stratum gave a statistic of 1.61 (p = 0.205), against 80.0 (p < 0.0001) for transformation itself (Section S1.3).

| **Age** | **Sex** | **Grade** | **Integrated diagnosis** | **Enhancement** | **Resection** | **Follow-up (y)** |
| --- | --- | --- | --- | --- | --- | --- |
| 58.9 | M | 3 | IDH-wildtype | absent | Biopsy | 1.4 |
| 37.8 | M | 2 | Astrocytoma, IDH-mutant | absent | GTR | 0.9 |
| 66.2 | M | 2 | Oligodendroglioma, IDH-mut 1p/19q | absent | STR | 3.5 |
| 76.7 | M | 2 | Oligodendroglioma, IDH-mut 1p/19q | absent | STR | 2.0 |
| 32.7 | M | 2 | Oligodendroglioma, IDH-mut 1p/19q | absent | STR | 1.1 |
| 76.0 | F | 2 | IDH-wildtype | absent | STR | 0.4 |

# S7. The contrast-enhancement read: criteria and rationale

A tumour was scored enhancing (sub-threshold) when both of the following held: (i) at least one focus of unequivocal post-contrast signal increase relative to the co-registered pre-contrast T1, visible on two or more contiguous slices; and (ii) a pattern that was neither ring-like nor solid-nodular, without central necrosis, a discrete enhancing nodule or multifocal enhancing deposits. A tumour meeting (i) but not (ii) met a radiological criterion for malignancy at diagnosis and was not eligible for the cohort, which is what keeps the baseline exposure and the transformation endpoint separable despite both being read from post-contrast T1 imaging.

Signal increase was judged against normal-appearing contralateral white matter in the same series rather than against an absolute intensity value, because the cohort spans 19 scanner models at 0.4–3.0 T over 23 years and absolute intensity thresholds are not transferable between them. The same argument applies to a volumetric threshold. Because segmentation was performed on FLAIR (or T2 where FLAIR was unavailable) and the whole-tumour mask was propagated across sequences, no mask of the enhancing sub-compartment exists, so enhancing volume fraction and maximum enhancing diameter cannot be reconstructed retrospectively for this cohort. Inter-reader agreement for this read is reported in Section S10 and Table S14.

# S8. Integrated histomolecular composition

Under WHO 2016 the cohort comprised 116 grade 2 and 39 grade 3 tumours. Reconstructed against the 2021 scheme, 59 patients had IDH-mutant astrocytoma, 47 IDH-mutant 1p/19q-codeleted oligodendroglioma and 42 IDH-wildtype tumours (Table S12). 1p/19q status was available in 149 of 155 patients, TERT-promoter testing in 51 and CDKN2A/B in 97; EGFR amplification and combined +7/−10 were not assessed and cannot be reconstructed, so a complete WHO CNS5 assignment is not attainable in this cohort. Within each represented entity every enhancing tumour transformed (11/11 astrocytoma, 9/9 oligodendroglioma, 11/11 IDH-wildtype) against 17/48, 8/38 and 15/31 of non-enhancing tumours. Because the enhancing, non-transforming cell is empty in every stratum, an unpenalised hazard ratio is not identifiable and stratum-specific effects are given as Firth penalised-likelihood and exact conditional estimates (Table S13). Under separation the point estimates remain unstable and the upper bounds uninformative; the interpretable features of that table are the lower bounds, which exclude unity in every stratum, and the consistency of direction across three biologically distinct entities.

**Supplementary Table S12.** Integrated histomolecular composition and transformation, reconstructed against the WHO CNS5 (2021) scheme from documented IDH and 1p/19q status.

| **Integrated diagnosis (WHO 2021 reconstruction)** | **n** | **Transformed** | **Did not transform** | **Enhancing: MT / n** | **Non-enhancing: MT / n** |
| --- | --- | --- | --- | --- | --- |
| Astrocytoma, IDH-mutant | 59 | 28 | 31 | 11 / 11 | 17 / 48 |
| Oligodendroglioma, IDH-mutant 1p/19q-codeleted | 47 | 17 | 30 | 9 / 9 | 8 / 38 |
| IDH-wildtype | 42 | 26 | 16 | 11 / 11 | 15 / 31 |
| IDH-mutant, 1p/19q not available | 3 | 3 | 0 | — | — |
| IDH status indeterminate | 4 | 3 | 1 | — | — |
| Molecular testing coverage | 1p/19q 149/155 | TERT 51/155 | CDKN2A/B 97/155 | EGFR not assessed | +7/−10 not assessed |

**Supplementary Table S13.** Firth penalised-likelihood and exact conditional estimates of the odds of ever transforming, by stratum. Every stratum shows quasi-complete separation (no enhancing tumour remained transformation-free), so only the lower confidence bounds are interpretable. ∞ denotes an unbounded exact estimate. The p column is Fisher’s exact test; Table S7 reports the penalised likelihood-ratio p for the same full-cohort model.

| **Stratum** | **Enhancing MT / n** | **Non-enhancing MT / n** | **Firth OR (95 % CI)** | **Exact OR (95 % CI)** | **Fisher p** |
| --- | --- | --- | --- | --- | --- |
| Full cohort | 33 / 34 | 44 / 121 | 38.9 (9.76–354) | 56.6 (8.8–2379) | 2.6 × 10⁻¹¹ |
| IDH-mutant | 22 / 22 | 26 / 87 | 104 (13.6–13446) | ∞ (11.4–∞) | 4.5 × 10⁻¹⁰ |
| IDH-wildtype | 11 / 11 | 15 / 31 | 24.5 (2.8–3242) | ∞ (2.1–∞) | 0.0027 |
| Astrocytoma, IDH-mutant | 11 / 11 | 17 / 48 | 41.4 (4.9–5433) | ∞ (3.9–∞) | 7.7 × 10⁻⁵ |
| Oligodendroglioma | 9 / 9 | 8 / 38 | 68.2 (7.4–9128) | ∞ (5.6–∞) | 1.8 × 10⁻⁵ |
| WHO grade 2 only | 18 / 19 | 29 / 97 | 28.6 (6.8–267) | 41.0 (5.9–1783) | 1.1 × 10⁻⁷ |
| WHO grade 3 only | 15 / 15 | 15 / 24 | 19.0 (2.1–2529) | ∞ (1.6–∞) | 0.0069 |
| Excl. WHO 2021 molecular GBM | 28 / 29 | 34 / 105 | 39.4 (9.7–362) | 56.9 (8.7–2423) | 1.2 × 10⁻¹⁰ |

# S9. Landmark analyses and E-values

Because much of the full-cohort association reflects tumours already transforming at diagnosis, a prespecified landmark series conditions on transformation-free survival at 3, 6 and 12 months, excluding patients whose event or follow-up ended before the landmark and restarting the clock there. The CE hazard ratio was 7.41 (95 % CI 3.26–16.85) at three months (n = 128, 52 events), 5.65 (2.23–14.31) at six months (n = 119, 47 events) and 6.33 (2.47–16.19) at twelve months (n = 111, 44 events). Figure S5 shows the same comparison without a model, as Turnbull non-parametric cumulative-incidence estimates with the intervals the interval-censored data cannot resolve shown explicitly.

E-values (VanderWeele and Ding) were computed for the landmark, post-surgical and histology-restricted estimates by converting each hazard ratio to an approximate risk ratio for a common outcome. For the six-month landmark the E-value was 5.84 for the point estimate and 2.87 for the confidence limit; for the three- and twelve-month landmarks 7.01/3.91 and 6.31/3.13; for the post-surgical model 4.79/2.81; and for the histologically confirmed endpoint 2.83/1.13. An unmeasured confounder would therefore have to be associated with both enhancement and transformation by a risk ratio of at least 5.8, above and beyond the measured covariates, to explain the six-month estimate away.

**Supplementary Figure S5 | Turnbull non-parametric cumulative incidence of malignant transformation by baseline contrast enhancement.** (A) Full cohort, time from diagnosis. (B) Post-surgical cohort, time from the first operation. Solid segments are intervals in which the non-parametric maximum-likelihood estimate is determined; shaded rectangles mark the regions the interval-censored data cannot resolve, within which the curve may take any monotone path. No proportional-hazards or parametric assumption is used.

# S10. Observation process, reader agreement and internal validation

Two reviewers with four and five years of experience, not involved in the original assessment, re-scored the baseline and transformation examinations independently and blinded to follow-up, to outcome and to one another (Table S14). Agreement on the exposure variable was complete in the study cohort (46/46, κ = 1.000) and 84/85 across all re-scored examinations (κ = 0.976).

Bracket widths and imaging intensity by exposure, molecular status and treatment era are given in Table S15. The median bracket width was 14.4 months (IQR 0.6–54.1) overall: 0.4 months for events preceding the first operation and 45.1 months for those following it. Brackets were narrower in enhancing patients (0.5 vs 47.7 months, p < 0.001), but the number of pre-treatment MRI examinations was identical in the two groups (median 3, p = 0.61) and the difference in time to the first operation was ten days (22 vs 32 days, p = 0.016). The difference in bracket width follows from the timing of events rather than from surveillance behaviour: enhancing tumours transformed within weeks of diagnosis, so their brackets are anchored at zero and close almost immediately, whereas non-enhancing tumours transformed years later and after surgery. Because L is anchored to a fixed clinical landmark and not to the most recent negative examination, denser imaging cannot narrow a bracket; it can only bring R forward. Rounding all brackets onto a common 6- or 12-month grid, which removes any residual advantage of denser imaging, reduced the CE hazard ratio by 17 % and 28 % respectively, with the confidence interval excluding unity throughout. Only one enhancing patient did not transform, so the surveillance experience of enhancing non-transformers cannot be characterised; this is stated in the Discussion.

**Supplementary Table S14.** Inter-reader agreement. Two reviewers with four and five years of experience, not involved in the original assessment, re-scored the baseline and transformation examinations independently, blinded to follow-up, to outcome and to one another. The study cohort comprises the 46 re-scored examinations belonging to patients in the analysis; the remaining re-scored examinations are from the same institutional database and were included to increase the precision of the reliability estimate.

| **Read** | **Population** | **n** | **Agreement** | **Cohen’s κ (95 % CI)** |
| --- | --- | --- | --- | --- |
| Contrast enhancement at diagnosis (exposure variable) | study cohort | 46 | 46/46 (100 %) | 1.000* |
| Contrast enhancement at diagnosis (exposure variable) | all re-scored | 85 | 84/85 (98.8 %) | 0.976 (0.93–1.00) |
| New enhancement at the transformation examination | study cohort | 46 | 44/46 (95.7 %) | 0.913 (0.80–1.00) |
| New enhancement at the transformation examination | all re-scored | 85 | 83/85 (97.6 %) | 0.951 (0.88–1.00) |
| Radiological sign of malignancy, baseline | study cohort | 46 | 44/46 (95.7 %) | 0.898 (0.76–1.00) |
| Radiological sign of malignancy, transformation examination | study cohort | 46 | 42/46 (91.3 %) | 0.825 (0.66–0.99) |
| Reader vs the enhancement variable used in the analysis | study cohort | 46 | 45/46 (97.8 %) | 0.953 (0.86–1.00) |

** With complete agreement κ is 1.000 and its asymptotic variance is zero, so no confidence interval is defined; the exact one-sided 95 % lower bound on the agreement proportion is 93.7 %. The last row compares each reader against the enhancement variable used in the analysis and is identical for both readers.*

**Supplementary Table S15.** Interval width, imaging intensity and time to first operation, by exposure, molecular status and treatment era. Bracket width is R − L in months. p values are from the Mann–Whitney U test.

| **Stratum** | **n** | **Events** | **Bracket width, months, median (IQR)** | **p** |
| --- | --- | --- | --- | --- |
| All events | 155 | 77 | 14.4 (0.6–54.1) | — |
| before the first operation | — | 27 | 0.4 (0.2–1.1) | — |
| after the first operation | — | 50 | 45.1 (12.3–77.3) | — |
| Non-enhancing | 121 | 44 | 47.7 (17.9–80.2) | < 0.001 |
| Enhancing | 34 | 33 | 0.5 (0.2–4.0) |  |
| IDH-mutant | 109 | 48 | 43.5 (1.0–80.2) | 0.023 |
| IDH-wildtype | 42 | 26 | 5.0 (0.4–18.4) |  |
| Diagnosis 1998–2009 | 53 | 35 | 43.6 (4.0–105.7) | 0.002 |
| Diagnosis 2010–2021 | 102 | 42 | 5.6 (0.4–31.0) |  |

**Supplementary Table S15 (continued).** Imaging intensity by exposure status.

| **Measure** | **Non-enhancing** | **Enhancing** | **p** |
| --- | --- | --- | --- |
| Pre-treatment MRI examinations, median (IQR) | 3 (2–3) | 3 (2–3) | 0.61 |
| Diagnosis to first operation, days, median (IQR) | 32 (15–91) | 22 (15–36) | 0.016 |
| Surveillance among non-transformers, years | 4.7 (2.0–10.0) | n = 1; 6.1 | — |
| Bracket coarsened to a 6-month grid: CE HR | — | −17 % vs primary | < 0.001 |
| Bracket coarsened to a 12-month grid: CE HR | — | −28 % vs primary | < 0.001 |

Internal validity of the primary Cox counting-process model was assessed by Harrell’s bootstrap-optimism procedure and its calibration against the non-parametric Turnbull estimator (Table S16). Discrimination is good and the linear predictor is close to correctly scaled, but absolute risk is systematically over-predicted in non-enhancing tumours, so the model is not proposed for individual absolute-risk prediction.

**Supplementary Table S16.** Calibration and time-dependent prediction error of the primary Cox counting-process model. Predicted five-year risks are compared with the non-parametric Turnbull estimate in the same stratum; Brier scores are inverse-probability-of-censoring weighted and compared with a marginal (covariate-free) reference.

| **Measure** | **Result** |
| --- | --- |
| Calibration slope, apparent | 1.00 |
| Calibration slope, bootstrap-corrected (300 resamples) | 0.92 (0.67–1.20) |
| Harrell C-index, apparent / optimism / corrected | 0.847 / 0.018 / 0.83 |
| CE hazard ratio, apparent / bias-corrected | 12.1 / 8.5 |
| 5-year risk, non-enhancing grade 2: predicted vs Turnbull | 0.55 vs 0.27 |
| 5-year risk, non-enhancing grade 3: predicted vs Turnbull | 0.91 vs 0.62 |
| 5-year risk, enhancing grade 2: predicted vs Turnbull | 1.00 vs 0.93 |
| 5-year risk, enhancing grade 3: predicted vs Turnbull | 1.00 vs 1.00 |
| Brier score at 2 / 5 / 10 years, model | 0.204 / 0.243 / 0.251 |
| Brier score at 2 / 5 / 10 years, marginal reference | 0.228 / 0.250 / 0.255 |

Note on the two prespecified radiomic features. GLDM Dependence Variance on FLAIR quantifies the variability of local voxel-neighbourhood dependence within the tumour; high values indicate a spatially inconsistent infiltrative pattern rather than uniform infiltration. GLSZM Large Area Low Gray Level Emphasis on T1c is elevated when large contiguous zones of low post-contrast signal are present, corresponding to sizeable non-enhancing, presumably low-perfusion or microcystic regions. Neither feature reached credibility under the regularised horseshoe prior, and both were collapsed by the native interval-censored Fine–Gray model, so these interpretations are offered as context for the features carried forward from prior stability selection and not as findings of this analysis.
